# Could signal peptide complement C1r/C1s, Uegf, and Bmp1, and epidermal growth factor-containing protein 1 be a therapeutic target in the pathogenesis of preeclampsia?

**DOI:** 10.1590/1806-9282.20231027

**Published:** 2024-03-04

**Authors:** Kenan Toprak, Zafer Yıldız, Selim Akdemir, Kamil Esen, Rahime Kada Düken

**Affiliations:** 1 Harran University, Faculty of Medicine, Department of Cardiology – Şanlıurfa, Turkey.; 2 Harran University, Faculty of Medicine, Department of Obstetrics and Gynecology – Şanlıurfa, Turkey.; 3 Siverek State Hospital, Department of Obstetrics and Gynecology – Şanlıurfa, Turkey.

**Keywords:** Preeclampsia, SCUBE1, Biomarkers

## Abstract

**OBJECTIVE:**

Determination of biomolecules that play a role in the etiopathogenesis of preeclampsia and their application as therapeutic targets may increase surveillance in this patient group. The aim of this study was to investigate the relationship between signal peptide complement C1r/C1s, Uegf, and Bmp1, and epidermal growth factor-containing protein 1, a marker of endothelial dysfunction and platelet activation, and the development of preeclampsia.

**METHODS:**

In this observational cross-sectional study conducted between April 2021 and December 2022, 73 consecutive pregnant women with preeclampsia and 73 healthy pregnant women were included. Blood samples were taken from all patients with preeclampsia to measure signal peptide complement C1r/C1s, Uegf, and Bmp1, and epidermal growth factor-containing protein 1 levels at the time of hospitalization. Excluded from the study were pregnant women with certain medical conditions or treatments, and the signal peptide complement C1r/C1s, Uegf, and Bmp1, and epidermal growth factor-containing protein 1 levels of the groups were compared according to the development of preeclampsia.

**RESULTS:**

Signal peptide complement C1r/C1s, Uegf, and Bmp1, and epidermal growth factor-containing protein 1 levels were significantly higher in the preeclampsia group than in the controls (p<0.001). In multivariate analysis, signal peptide complement C1r/C1s, Uegf, and Bmp1, and epidermal growth factor-containing protein 1 was determined as an independent predictor for preeclampsia (OR: 1.678, 95%CI 1.424–1.979, p<0.001). Receiver operating characteristic curve analysis showed that the best cutoff value of signal peptide complement C1r/C1s, Uegf, and Bmp1, and epidermal growth factor-containing protein 1 at 3.25 ng/mL predicted the development of preeclampsia with 71% sensitivity and 68% specificity (area under the curve, 0.739; 95% confidence ınterval (95%CI), 0.681–0.798, p<0.001).

**CONCLUSION:**

Signal peptide complement C1r/C1s, Uegf, and Bmp1, and epidermal growth factor-containing protein 1 is significantly elevated in pregnant women with preeclampsia compared with healthy controls.

## INTRODUCTION

Preeclampsia is one of the most important causes of maternal and perinatal mortality and morbidity all over the world^1^. Preeclampsia is a serious pregnancy-specific hypertensive disease that presents with various organ failures, especially dysfunction of the kidneys, liver, and lungs^2^. Currently, the only known definitive treatment for preeclampsia is to terminate the pregnancy and deliver the newborn^3^, ^4^. Preeclampsia occurs in approximately 3–6% of all pregnancies, with an incidence 1.5–2 times higher in first pregnancies^5^. Generalized vasospasm, endothelial dysfunction, and secondary decreased organ perfusion with the activation of the coagulation cascade have been implicated in the pathogenesis of preeclampsia^6^. Maternal comorbidities closely associated with endothelial dysfunction and thrombotic complications such as chronic kidney disease, hypertension, and obesity play an important role in the etiopathogenesis of preeclampsia, and there is a lot of evidence that endothelial disease, the underlying mechanism of preeclampsia, is not limited to pregnancy but increases cardiovascular risk in later life^7^.

Although many comprehensive studies have been carried out in recent years to understand the pathogenesis of preeclampsia, the underlying pathogenesis still remains unclear^4^, ^8^, ^9^. It is claimed that these mechanisms play a role in the basic etiopathogenesis of preeclampsia, mainly related to endothelial dysfunction^10^. Due to the release of placental factors into the systemic circulation at the end of poor placental perfusion, triggering systemic inflammation, vascular endothelial dysfunction, oxidative stress, and platelet activation, signs of increased blood pressure, proteinuria, and hypercoagulation develop, and the clinical picture of preeclampsia emerges^11^.

Signal peptide complement C1r/C1s, Uegf, and Bmp1 and epidermal growth factor-containing protein 1 (SCUBE1) are recently identified cell surface proteins that can be expressed and secreted during early embryogenesis^12^. SCUBE1 is predominantly stored in the alpha granules of inactive platelets and endothelial cells^13^. After platelet activation, its expression increases and migrates toward the cell surface and is released into the circulation as small soluble particles. These circulating particles are considered a platelet activation marker because they increase platelet-platelet adhesion and agglutination in thrombotic conditions^13^. In addition, it is accepted as a marker of endothelial damage and vascular biology because its levels increase in the circulation in conditions associated with acute endothelial damages such as acute coronary syndrome, ischemic stroke, and hypertensive crisis^14^- ^16^. We thought that there may be a causal relationship between SCUBE1 and preeclampsia, as the main presentations of preeclampsia, such as endothelial damage, hypertensive state, and prothrombotic environment, are pathophysiological conditions in which SCUBE1 also plays an active role, as mentioned before. In addition, the fact that aspirin, which is an antithrombotic agent, is the only proven treatment method in preeclampsia prophylaxis today strongly suggests the active role of SCUBE1, which is a platelet activation marker, in preeclampsia^17^. Thus, this biomarker may be a potential diagnostic marker and therapeutic target for preeclampsia.

As there are no enough data in the literature, our aim in this study was to compare the levels of SCUBE1, which is a marker of endothelial dysfunction and platelet activation, between healthy pregnant women and pregnant women with preeclampsia, and also to investigate the relationship between SCUBE1 levels and the severity of preeclampsia.

## METHODS

### Study setting and population

This observational cross-sectional study included 73 consecutive pregnant women hospitalized for preeclampsia between April 2021 and December 2022 and 73 healthy normotensive pregnant women matched by gestational age. Pregnant women with diabetes, history of chronic hypertension, liver disease, chronic kidney failure, history of thromboembolic event or thrombophilic disease, active infection, multiple pregnancies, having had preeclampsia before, HELLP syndrome, pregnant women using antiaggregant or anticoagulant, and those whose written consent could not be obtained for the study were excluded. Blood samples were taken from all patients with preeclampsia to measure SCUBE1 levels at the time of hospitalization. SCUBE1 levels were compared between the gestational age-matched healthy control group and the preeclampsia group. The sample size for this study was estimated based on common assumptions for a two-sample t-test with a significance level (alpha) of 0.05 and a power (1 - beta) of 0.80. We assumed a moderate effect size (Cohen’s d=0.5) for the difference in SCUBE1 levels between the preeclampsia group and the healthy control group. An estimated sample size of approximately 67 participants in each group (preeclampsia and healthy control) would be required to detect a significant difference in SCUBE1 levels. Therefore, the estimated sample size was found to be adequate to detect significant differences in SCUBE1 levels between the two groups, considering the stated assumptions.

This study was conducted in line with the principles of the Declaration of Helsinki. The study was approved by the local ethics committee (Date: 21.03.2021, No. 21.02.01). Informed consent was obtained from all participants.

### Clinical definitions

Preeclampsia was defined as the presence of one or more of the following new-onset conditions in pregnant women diagnosed with hypertension after 20 weeks of gestation: (1) proteinuria; (2) maternal organ dysfunction, including (a) renal failure (creatinine>90 μmol/L; 1 mg/dL), (b) liver involvement (elevated transaminases with or without right upper quadrant or epigastric abdominal pain), (c) neurological complications (including eclampsia, altered mental status, blindness, stroke, hyperreflexia with clonus, severe headaches with hyperreflexia, and persistent visual scotomata), and (d) hematological complications (thrombocytopenia with a platelet count below 150,000/dL, disseminated intravascular coagulation, and hemolysis); and (3) uteroplacental dysfunction (such as fetal growth retardation and abnormal umbilical artery Doppler wave)^18^.

### Laboratory analysis

Venous blood samples were obtained from pregnant patients with preeclampsia shortly after hospitalization and during routine polyclinic examinations from the control group matched for gestational age. Plasma and serum samples were obtained after centrifugation at 2750×g for 10 min. Routine biochemical analyses were performed on blood samples. Serum samples for SCUBE1 analysis were frozen and stored at -20°C until assayed. SCUBE1 levels were measured using the commercial enzyme-linked immunosorbent assay (ELISA) kits (Human SCUBE1 ELISA kit: Aviva Systems Biology, San Diego, USA). The ELISA kit range was at a concentration of 0.156–10 ng/mL. The results are presented in ng/mL for SCUBE1. The mean coefficients of variation (CV) ranged from intra-assay: CV<6.5% to inter-assay: CV<9.5%.

### Statistical analysis

Statistical Program for Social Sciences 26 (IBM SPSS, Chicago, IL, USA) was used for statistical calculations. The Kolmogorov-Smirnov test was used to determine whether the data fit the normal distribution. Continuous variables that fit the normal distribution were expressed as means±standard deviation (SD), and those that did not fit the normal distribution were expressed as median with interquartile range (IQR). Comparisons between subjects with preeclampsia and the control group were analyzed using the Mann-Whitney U test and independent-sample t-test where appropriate. The chi-square test was applied to categorical variables. Multivariate regression analyses were performed to determine the independent predictors of preeclampsia. The selection of independent variables for logistic regression analysis was guided by a stepwise variable selection approach. The “forward selection” method was used, in which variables were added to the model one by one according to their statistical significance. At each step, the variable with the lowest p-value was included and the goodness of fit of the model was evaluated. Variables that did not contribute significantly to the model or improve its fit were not included. Receiver operating characteristic (ROC) curve analysis was performed to determine the optimum cutoff value of SCUBE1 levels, and we employed the ROC curve analysis to explore the potential utility of SCUBE1 as a biomarker for distinguishing between preeclampsia and healthy pregnancies. Two-tailed p<0.05 were considered statistically significant.

## RESULTS

**Figure 1.**
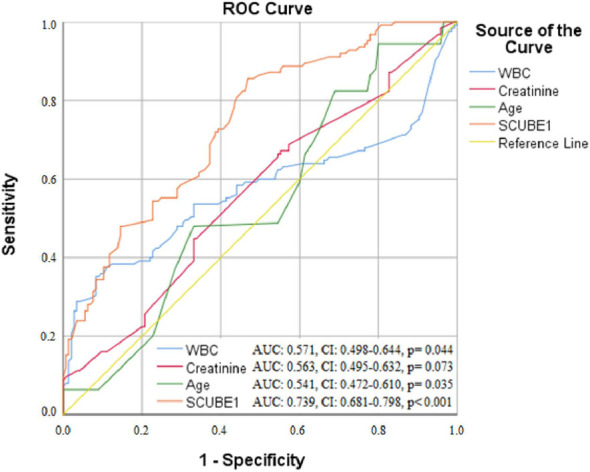
Receiver operating characteristic curve analysis showed that the best cutoff value of signal peptide complement C1r/C1s, Uegf, and Bmp1 and epidermal growth factor-containing protein 1 at 3.25 ng/mL predicted the development of preeclampsia with 71% sensitivity and 68% specificity (area under the curve, 0.739; 95% confidence interval, 0.681–0.798, p<0.001). Also, when signal peptide complement C1r/C1s, Uegf, and Bmp1 and epidermal growth factor-containing protein 1 and other predictors (i.e., white blood cell, creatinine, and age) were compared pairwise, the predictive power of signal peptide complement C1r/C1s, Uegf, and Bmp1 and epidermal growth factor-containing protein 1 to predict preeclampsia was stronger than the other predictors (p<0.001 for all).

A comparison of demographic, clinical, and laboratory parameters of pregnant women with preeclampsia and the control group is given in Table 1. The mean age of the preeclampsia group was significantly higher than the control group (33.5 [4.9] vs 32.1 [5.9], p=0.039). While hematocrit levels were significantly lower in the preeclampsia group than in the control group (p=0.012), creatinine, white blood cell (WBC), and C-reactive protein (CRP) were higher (p=0.016, p=0.039, and p=0.016, respectively). SCUBE1 levels were significantly higher in the preeclampsia group than in the control group (4.63 [1.90] vs 3.09 [1.70]; p<0.001) (Table 1). In multivariate analysis, age (odds ratio [OR]: 1.056, 95% confidence interval (CI): 1.004–1.110, p=0.035), creatinine (OR: 1.280, 95%CI 1.059–1.569, p=0.041), WBC (OR: 1.152, 95%CI 1.043–1.273, p=0.005), and SCUBE1 (OR: 1.678, 95%CI 1.424–1.979, p<0.001) were determined as independent predictors for preeclampsia (Table 2). ROC curve analysis showed that the best cutoff value of SCUBE1 at 3.25 ng/mL detected the development of preeclampsia with 71% sensitivity and 68% specificity (area under the curve (AUC), 0.739; 95%CI 0.681–0.798, p<0.001). Also, when SCUBE1 and other predictors (i.e., WBC, creatinine, and age) were compared pairwise, the predictive power of SCUBE1 to predict preeclampsia was stronger than the other predictors (p<0.001 for all) (Figure 1).

**Table 1.** Comparison of demographic characteristics and hematological and biochemical parameters of the study groups

Variables	Control group (n=73)	Preeclampsia group (n=73)	p-value
Age (years)	32.1±5.9	33.5±4.9	0.039
Body mass index (kg/m^2^)	29.1±1.9	29.3±3.4	0.486
Gravidity (n)	4.8±3.4	5.0±2.5	0.550
Parity (n)	3.3±2.6	3.3±2.8	0.994
Gestational age at blood sampling (weeks)	35.04±3.61	35.08±3.61	0.216
Hemoglobin (g/dL)	10.77±0.55	10.75±0.41	0.735
Hematocrit (%)	35.5 (33.0–37.0)	34.5 (32.9–36.0)	0.012
Blood urea nitrogen (mg/dL)	38.0 (32.0–44.9)	36.0 (27.0–44.9)	0.101
Uric acid (mg/dL)	5.2 (4.4–6.1)	5.3 (4.3–6.1)	0.605
Creatinine (mg/dL)	0.8 (0.7–0.9)	0.8 (0.7–1.0)	0.016
White blood cell (×1000/mm^3^)	6.52 (5.29–8.20)	7.80 (4.48–10.45)	0.039
C-reactive protein (mg/dL)	0.50 (0.18–1.00)	0.64 (0.32–1.50)	0.016
SCUBE1 (ng/mL)	3.09±1.70	4.63±1.90	<0.001
Newborn’s birth weight (g)	2976±413	2672±452	0.093
Gestational age at delivery	38 (37–40)	37 (35–38)	0.109

Values are mean±SD, n (%), or median (interquartile range) unless otherwise stated.

**Table 2.** Univariate and multivariate regression analysis to identify independent predictors of preeclampsia

	Univariate analysis		Multivariate analysis	
	OR (95%CI)	p-value	OR (95%CI)	p-value
Age	1.046 (1.002–1.091)	0.040	1.056 (1.004–1.110)	0.035
Hematocrit	0.996 (0.980–1.012)	0.612	0.988 (0.967–1.009)	0.266
Creatinine	1.976 (1.098–2.496)	0.006	1.280 (1.059–1.569)	0.041
White blood cell	1.155 (1.061–1.256)	0.001	1.152 (1.043–1.273)	0.005
SCUBE1	1.604 (1.383–1.859)	<0.001	1.678 (1.424–1.979)	<0.001

## DISCUSSION

In this study, we found that SCUBE1 was significantly higher in preeclamptic pregnant women than in the healthy control group. In addition, we found SCUBE1 significantly higher in the severe preeclampsia group than in the mild preeclampsia group, indicating that SCUBE1 may also be closely related to the severity of preeclampsia. Furthermore, we demonstrated that SCUBE1 may be an independent predictor of preeclampsia.

Preeclampsia is a pregnancy-specific disease that significantly increases maternal and perinatal mortality and morbidity. This disease causes the fetus to be premature and the risk of cardiovascular disease increases significantly in the long term in the mother^19^. Preeclampsia is manifested by new-onset maternal hypertension and often proteinuria after the 20th week of pregnancy and may result in hepatic, renal, and cerebral end-organ damage, and nowadays almost the only treatment is delivery of the placenta and fetus^4^, ^5^. Although many pathophysiological mechanisms and risk factors have been identified in the etiopathogenesis of preeclampsia, its pathophysiology is still not fully elucidated^5^. It is suggested that unsuccessful transformation of uterine spiral arteries by trophoblasts in early pregnancy leads to poor placentation, causing hypoperfusion in the placental bed and fetal tissues, and as a result, placental factors released from the placenta into the maternal circulation lead to endothelial dysfunction, which is the main pathophysiology of preeclampsia^20^. This endothelial dysfunction creates a prothrombogenic predisposition and also increases the risk of thrombotic complications in these patients^21^.

Many endothelial functions are mediated by proteins selectively expressed on the endothelial surface, such as SCUBE1^22^. SCUBE1 shares homology with proteins involved in thrombotic processes such as fibrillin, thrombomodulin, and protein C^22^. In addition to endothelial cells, it is stored in the alpha granules of platelets and actively participates in platelet adhesion and aggregation; therefore, it is considered a marker of vascular biology and platelet activation^14^. Studies have shown that SCUBE1 plays an active role in diseases such as acute coronary syndrome, ischemic stroke, acute mesenteric Ischemia, and hypoxic renal damage that present with endothelial dysfunction and thrombotic processes^15^. Studies have found a causal relationship between SCUBE1 and hypertension, which is one of the major risk factors for the development of preeclampsia^23^. In another study, SCUBE1 levels were found to be significantly higher in case of hypertensive crisis, and preeclampsia is actually a hypertensive crisis specific to pregnant women^17^. The fact that endothelial dysfunction and prothrombotic processes that trigger these comorbidities are also involved in the basic pathophysiological processes that trigger preeclampsia supports the causal relationship between preeclampsia and SCUBE1, an endothelial dysfunction, vascular damage, and thrombosis marker. In another study, it was shown that SCUBE1 could be a marker that could indicate placental dysfunction in patients with gestational diabetes^24^. Previous studies have shown a close relationship between preeclampsia and gestational diabetes and its associated placental dysfunction, and these results support the association between SCUBE1 and preeclampsia^25^.

In conclusion, our findings suggest that SCUBE1 may serve as a valuable biomarker and predictor for the development and severity of preeclampsia. Identifying elevated SCUBE1 levels in pregnant women could potentially enhance the surveillance and early intervention in this patient group, ultimately contributing to improved maternal and perinatal outcomes. Furthermore, the role of SCUBE1 in the pathophysiology of preeclampsia highlights its potential as a therapeutic target. Future research, including larger randomized controlled studies, should aim to confirm these results and explore the clinical implications and therapeutic applications of SCUBE1 in the prediction and management of preeclampsia.

### Limitations

Our study has some limitations. First of all, our study population was relatively small and it was an observational study. Second, only a single third-trimester measurement was taken for SCUBE1 in our study, and serial measurements were not taken before and after pregnancy. Third, long-term follow-up was not performed for thrombotic complications and cardiovascular diseases. The addition of serial measurements and long-term follow-up would have made the study more valuable. Fourth, markers such as the sFlt-1/PLGF ratio that were previously proven in the development of preeclampsia were not evaluated in our study. Fifth, our study is a cross-sectional study and therefore does not reveal a definitive relationship between SCUBE1 and preeclampsia. Finally, the ethnic diversity of the sample may have affected the results, although we included consecutive patients in the study. Larger randomized controlled studies are needed to confirm our results.

## CONCLUSION

In our study, we detected higher levels of SCUBE1 in pregnant women with preeclampsia compared with healthy controls. Furthermore, we showed that SCUBE1 may be an independent predictor for the development of preeclampsia. These results indicate that the development of preeclampsia, in which endothelial dysfunction is a hallmark in etiopathogenesis, may be mediated by SCUBE1, an endothelial dysfunction and vascular biology marker. In this context, SCUBE1 may be a marker that can help explain the pathogenesis of preeclampsia.
